# Substitutional Analysis of Orthologous Protein Families Using BLOCKS

**DOI:** 10.6026/97320630013001

**Published:** 2017-01-19

**Authors:** Parth Sarthi Sen Gupta, Shyamashree Banerjee, Rifat Nawaz Ul Islam, Vishma Pratap Sur, Amal K. Bandyopadhyay

**Affiliations:** 1 Department of Biotechnology, The University of Burdwan, Golapbag, Burdwan, 713104, West Bengal, India;; 2 Indian Institute of Chemical Biology, Animal House (IICB), Kolkata, West Bengal, India;

**Keywords:** evolution, substitution, non-conservative, conservative, hetero-pairs, divergence rate

## Abstract

Orthologous proteins, form due to divergence of parental sequence, perform similar function under different environmental and biological conditions. Amino acid changes at locus specific positions form hetero-pairs whose role in BLOCK evolution is yet to be understood. We involve eight protein BLOCKs of known divergence rate to gain insight into the role of hetero-pairs in evolution. Our procedure APBEST uses BLOCK-FASTA file to extract BLOCK specific evolutionary parameters such as dominantly used hetero-pair (D), usage of hetero-pairs (E), non-conservative to conservative substitution ratio (R), maximally-diverse residue (MDR), residue (RD) and class (CD) specific diversity. All these parameters show BLOCK specific variation. Conservative nature of D points towards restoration of function of BLOCK. While E sets the upper-limit of usage of hereto-pairs, strong correlation of R with divergence-rate indicates that the later is directly dependent on non-conservative substitutions. The observation that MDR, measure of positional diversity, occupy very limited positions in BLOCK indicates accommodation of diversity is positionally restricted. Overall, the study extract observed hetero-pair related quantitative and multi-parametric details of BLOCK, which finds application in evolutionary biology.

## Background

Homologous proteins, emerged due to speciation event, are structurally and functionally similar [1]. Evolution accommodates changes in these sequences. Amino acid changes are mostly achieved by substitution, deletion and insertion mechanisms [2], of which earlier is the result of accumulation of changes at locus specific positions. In evolution, two types of substitutions namely conservative and non-conservative occur of which most of the later changes are deleterious. Thus these are eventually eliminated through purifying selection. Beneficial ones (both conservative and non-conservative) are restored in sequence population and thus contribute to species differentiation [3]. Comparison among homologous sequences of database reveals sequences of closely related species (e.g. human and mouse) are more similar than that of distantly related species (human vs. bacteria). When homologous positions (column-wise in a BLOCK) are fixed, it would be seen that each of these positions bears characteristic details. While some are invariant, other is either conservative or non-conservative type of substitutions [3]. Henikoff and Henikoff (1992) pioneered the concept of BLOCK of sequences. A BLOCK contains homologous sequences whose allelic positions are fixed. These types of BLOCKs of different level of sequence similarity were used to develop different series of average BLOSUM matrices [4].

The concept divergence rate has become an important tool in the assessment of mechanisms of diversification in sequence evolution [5]. Table values of divergence rates of few of these protein families are available [6,7]. Although different homologous proteins possess different divergence rate [7,8], for a given family, it is constant [9]. For example, Fibrino-peptide, a blood-clotting factor, has the highest and histone, a DNA binding protein, has the lowest divergence rate [7,8]. The variability in these rates are related to structural and functional requirements of these molecules [10]. In this aspect, great deals of studies and developments are available [6,7 and 9]. Understanding the mechanism of substitutions largely involve comparison of locusspecific positions [11], for its effect on physicochemical properties [12] and identity [13] or similarity [14]. Similarity or identity scores are used for pair-wise comparison of sequence that eventually helps their alignment, finding relatedness [14], obtaining functional significance and constructing phylogenetic trees [12,15]. Further sequence-based studies also include analyses and extraction of information from INDEL regions of alignment. It is an additive alternative to substitution-mechanism for understanding protein evolution [16]. While these studies have widened our understanding in different aspects of molecular evolution of protein sequences, the governing principles of evolution for homologous protein families in relation to acquired substitutions (i.e. the usage of observed hetero-pairs) still remain an enigma. Fundamental question concerning the non-conservative substitutions, as to how these are managed in these functionally similar proteins when they are known to be deleterious [3,17], remain to be answered.

In this work, we report results on SHPs (substitution-heteropairs) for eight protein BLOCKs of known divergence rate [6,7] to work out a general model of evolution of homologous proteins. We use APBEST for efficient extraction of BLOCK parameters (D, R, E, MDR, RD and CD). The study then shows the application of these parameters in relation to amino acid substitution of which the role of R and MDR are highlighted for the first time in this work. Overall our study extracts evolutionary parameters, the knowledge of which has potential application in understanding molecular evolution of homologous protein families.

## Methodology

### Collection of Data

A total of eight homologous protein families (Ubiquitin, Glyceraldehyde-3-phosphate dehydrogenase (G3PDH), Lactate dehydrogenase (LDH), Acid-protease, Hemoglobin, Ribonuclease, Somatotropin and Kappa-casein.) were taken in the present study. These families were chosen in such a way that their divergence rate give a wide coverage. For example Ubiquitin has 0.1% per 100/mYr and that for Kappa-casein is 33% per 100/mYr [6,7] . Family specific sequences were obtained from UNIPROT [18], database. Obtained sequences were then aligned using ClustalW2 [13], for each of the eight protein families.

### Preparation of BLOCK FASTA files

**Figure 1:**
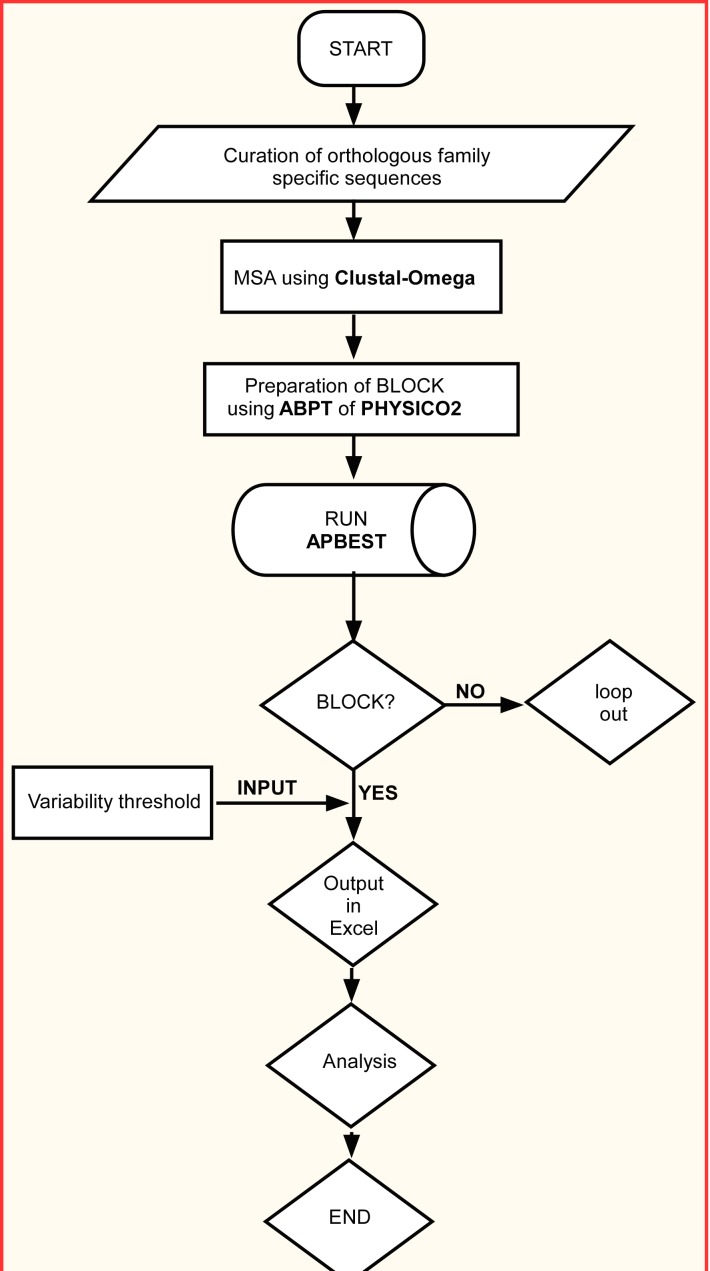
Flowchart describing methodology and operation of APBEST for extraction of analytical parameters from orthologous protein family.

BLOCK-FASTA files were prepared using automated block preparation tool (ABPT) of PHYSICO2 [19]. As the method involve manual step during removal of partial sequences, care was taken such that maximal sequence information is restored in the BLOCK. The BLOCK FASTA file thus produced was used as input for APBEST. An example input BLOCK FASTA file can be downloaded at (https://sourceforge.net/projects/apbest/files/). A flowchart starting from methodology to analysis using APBEST is shown in Figure 1.

### Analyses of BLOCK FASTA file and extraction of evolutionary parameters

**Figure 2:**
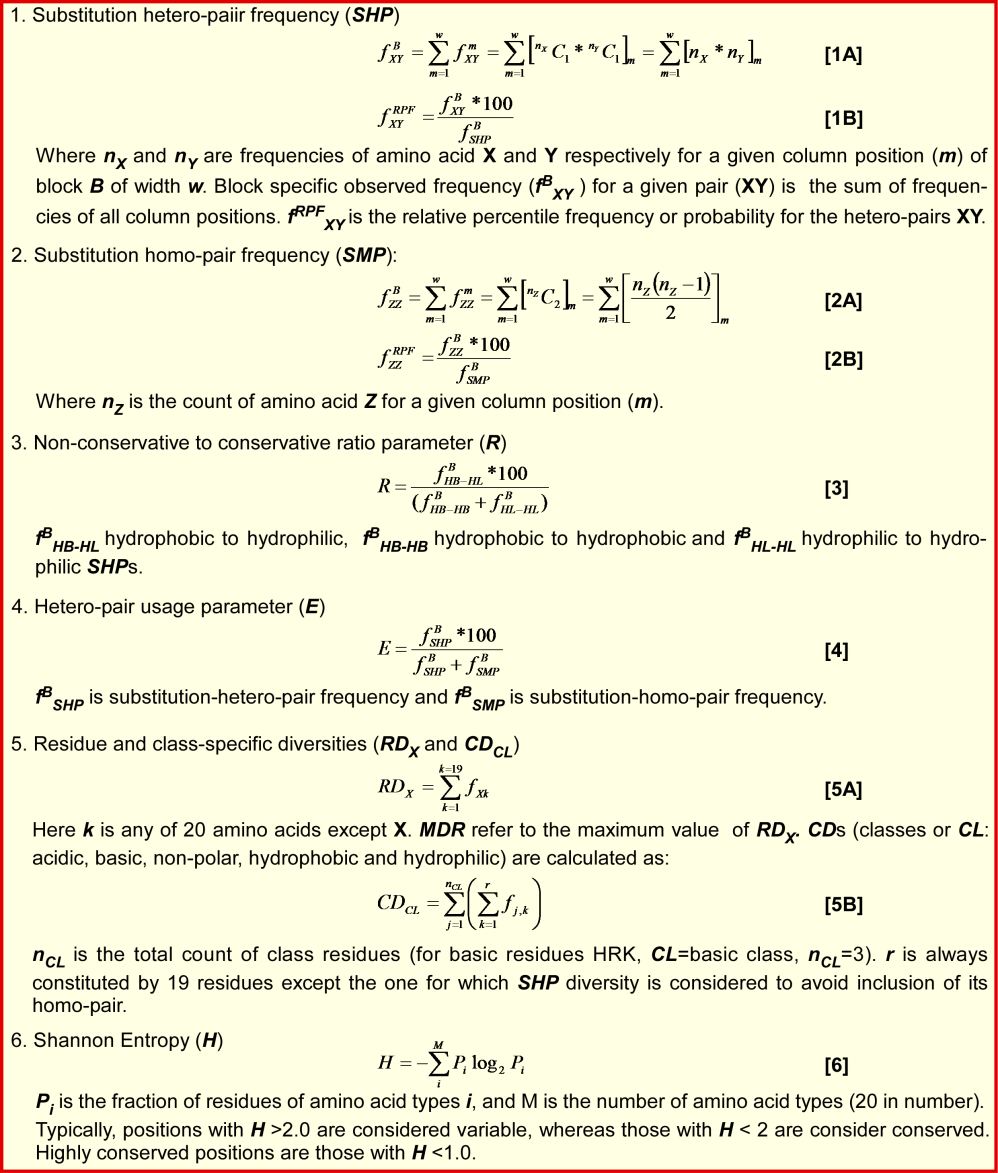
APBEST implemented equations and their clarity.

Analysis of BLOCK FASTA files was performed using in house procedure APBEST. The program is written in AWKprogramming- language and runs in CYGWIN-UNIX like environment. It is efficient, error free and user-friendly. A compact itemized (Item A through F) output is redirected in excel file. It is freely available at http://sourceforge.net/projects/APBEST/ for academic users. D, R, E, MDR, RD and CD parameters were computed using relevant observed frequency of substitutionhetero- pair (SHP) (Figure 2). BLOCK positions undergo different types of substitutions. Different positions of BLOCK are also assessed based on residue types. If there is only one type of amino acid in a given position then it is marked as invariant. If substituted then qualitatively positional substitutions are assessed as different categories such as hydrophobichydrophobic, hydrophilic-hydrophilic and hydrophobic to hydrophilic etc.

## Result and discussion

To explore evolutionary and functional significance of substitution-hetero-pairs (SHPs) for any given homologous protein family, we have analyzed eight homologous protein BLOCKs of known divergence rate [6, 7], (Table 1: second column) using APBEST. A representative output is available at https://sourceforge.net/projects/apbest/files/. It provides details of six different items (Item A through F). Items A to E compute quantitative results on substitutions. Item F provides qualitative and quantitative insight into the positional mutations and variability respectively. The study is a first time attempt to gain insight into the mechanism of substitution based on observed hetero-pairs and its diversity. It is worth noting here that, BLOSUM series of fundamental matrices made use of observed hetero-pair for the computation of odd-score [4]. However, their use in relation to the above is rare.

**Figure 3:**
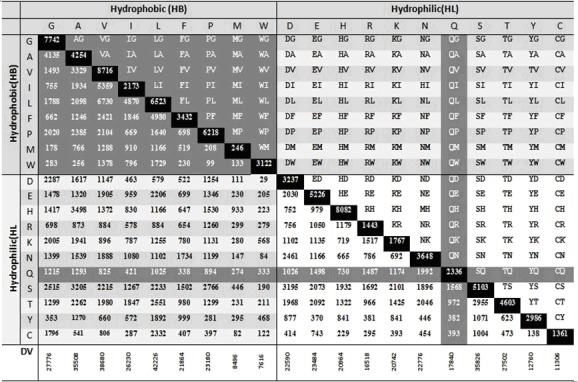
190 SHP types (upper-half of diagonal) and observed frequencies (lower-half of diagonal) are shown. Substitution-homo-pair frequencies (i.e. 20) are at the diagonal position. Both these types and their frequencies divided into three categories: a] HB-HB category: total 36 (upper dark shade), b] HL-HL category: total 55 (lower white shade) and HB-HL category: 99 in number (middle gray shade region). Residue Q is shown by gray-strip for explanation of the calculation of diversity of a given hetero-pair.

In the course of evolution, observed SHPs, the source of diversity in BLOCK, emerge in expense of homo-pairs in the ancestral protein. A total of 20 homo-pairs (diagonal) and 190 hetero-pairs (off-diagonal) participate in this process. BLOCK specific frequency parameters such as R, E and N, and diversities parameters such as RD, CD and MDR are presented in Table 1. Homo-pair and hetero-pair frequencies and types for a typical BLOCK are presented in Figure 3. Several points are noteworthy from Table 1 and Figure 3. First, type specific hetero-pair frequencies are seen to be non-identical for BLOCKs (Figure 3) and usage of hetero-pair (E) for different BLOCKs are seen to be different (Table 1: column 5). Second, dominantly used heteropair (D) is seen to be conservative in nature (Table 1: column 8). Third, residue (Table 1: column 6-7) and class-specific (Table 1: column 9-13) diversities (RD and CD respectively) also show BLOCK specific variation. Interestingly, type of MDR (Table 1: column 6; Frequency: 18 to 26) is more versatile than that of minimally diverse residue (Table 1: column 7; Frequency: 0 to 2). Finally, ratio parameters (R, E and N) also show BLOCK specific variation.

**Figure 4:**
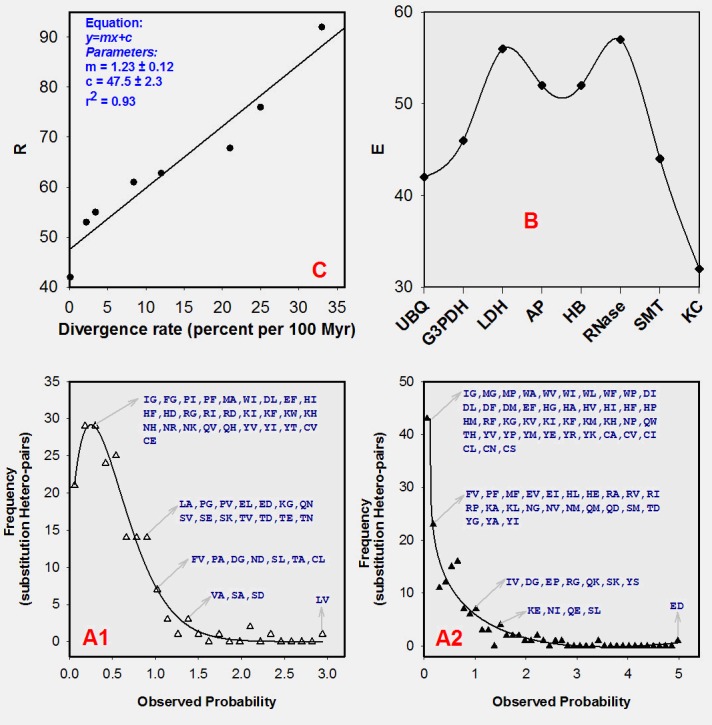
Plot of derived ratios (R and E) vs. divergence rate and observed hetero-pair frequency vs. probability range. Two typical frequency distributions are shown (Graph A1 and Graph A2) where the observed-data are fitted with Weibull distribution function. Used (E) fraction of hetero-pair is plotted against divergence rate (Graph B). Graph C, shows the correlation between R with divergence rate [6,7].

The fact that for a given BLOCK, individual SHP frequency varies from one another (Figure 3) and among BLOCKs, E also shows variation (Table 1: column 4), we have presented heteropair frequency against observed probability in Figure 4 (plot A1 and A2). It is seen in the figure that overall distribution pattern and region specific details of observed hetero-pair types vary greatly for BLOCKs. At low probability range, observed heteropair frequency is very high and non-selective. As we move towards higher probability range, the frequency and type of hetero-pair become narrower and selective. For example, at highest probability range, the sole and lone observed hetero-pairs are LV and ED for plot A1 and A2 respectively (Figure 4). It is worth noting here that both of these are conservative types with the former is hydrophobic and the later is hydrophilic.

In evolution, functionally similar sequences (BLOCK of homologous/Orthologous sequences) are the result of substitution in the parental one. While conservation of specific sequence positions as parental one (such as active site, binding site, protein core forming region etc) is the prerequisite for functionality, evolution demands substitutions (i.e. formation of SHPs) at homologous positions for environmental adaptation. At the same time, lethal substitutions may lead to the malfunctioning of proteins [3,17]. At this point, it is worth raising the question as to what are the lower and upper limits of usage of SHPs. To check this, we have plotted E for BLOCKs (Figure 4: Plot B). In principle, E varies between 0 and 1 (Figure 2; Equation 4). The former and the later indicate non-use and fulluse of SHP respectively. However, we see the observed lower and upper limit of E are 0.3 and 0.7 respectively. Interestingly kappa-casein, that possesses highest divergence rate (Table 1: column 1) shows lower E value (0.32). Similar is the case for Somatotropin. Thus, the parameter E is largely uncorrelated to the divergence rate.

Is there a BLOCK specific parameter that would correlates divergence rate? In Figure 4 (C) R is plotted and fitted against the divergence rates [6,17]. Notably, it is the ratio of nonconservative to conservative substitution (Figure 2; Equation 3). The plot shows that the parameter is positively and linearly correlated with divergence rate (correlation coefficient of 0.93). Such strong correlation of R and divergence rate indicates the former could be useful in the analysis of substitutions of orthologous protein families.

Many factors might affect BLOCK’s-positional divergence or diversity. Some of these factors are positional entropy (Shannon) [20], position specific physicochemical characteristics of BLOCKs. APBEST also computes some details of which few are listed in Table 2. Several points are noteworthy from the Table A] Majority (≥60%) of sequence positions in BLOCKs contains mixed type (HB+HL) amino acid. Thus, HB+HL-type dominates over others such as HB, PU+PC etc. b] All but hemoglobin and ribonuclease contains invariant-lines with highest for G3PDHBLOCK. Invariant-line does not evolve over time and are largely involves in the preservation of function of BLOCK as parental one. c] Shannon entropy is the measure of positional conservation [20]. A value ≤1.0 indicate highly conserved positions. Details of conserved positions are shown in Table 2 (column 4). Highest and lowest conservation is seen in the case of kappa-casein (65%) and ribonuclease (11%) respectively. At this point, it is worth mentioning that kappa-casein with highest divergence rate and highest R-value shows high positional conservation (64%; Shannon entropy≤1.0). This apparent contrast of high divergence rate and high conservation of kappa casein BLOCK could be resolved by the observation that non-conservative substitutions (determinant of divergence rate) occurs only at limited and unique BLOCK positions. Such limit might allow protein to use rest of the BLOCK positions for conservation to retain function.

## Conclusion

Analyses of 8 protein BLOCKs of known divergence rate shows BLOCK specific variation in the distribution pattern, hetero-pair frequency and parameters such as D, E and R, MDR, RD and CD. E is suitable for understanding usage limit of hetero-pairs and R is directly related with the divergence rate. Non-conservative substitution acts as determinant for the divergence rate. MDR not only contributes to class-specific-variability (CD-parameter) but also contributes to divergence rate. It populates only at limited BLOCK positions indicates the divergence utilizes limited portion of the total width of BLOCK. In other words, BLOCK with high conservation can still have high divergence. Such a novel strategy of limited yet unique use of positions for divergence is postulated for the purpose of incorporation of other important mechanisms of substitutions such as conservation. Taken together the procedure seems to have novel applications in substitution analysis of orthologous protein families.

## Conflict of Interest

Authors would like to declare no conflict of interest.

## Figures and Tables

**Table 1:** BLOCK specific quantitative parameters for SHPs as obtained by APBEST analysis

Name of Protein BLOCK	Divergence Rate*	Computed Ratio parameters			Residue diversity RD		€ Dominant pair (D)	Class specific diversity CD				
		R (%)	E (%)	N (%)	MDR or RDMAX	RDMIN		Acidic	Basic	Non-polar	Hydrophobic	Hydrophilic
Ubiquitin	0.1	42	42	18	K (18.8)	C (0.5)	IV (5.1)	30.5	34.8	49.8	50.7	78.6
G3PDH	2.2	53	46	1	V (21.2)	W (1.4)	IV (9.2)	23.3	24	36.2	69.8	64.9
LDH	3.4	55	56	0	V (20.9)	W (1.8)	IV (7.6)	18.8	21.9	44	69.4	66.1
Acid-protease	9	62	52	2	S (23.5)	C (1.6)	IV (4.7)	16.5	13.5	57.3	68.6	69.6
Hemoglobin	12	63	52	0	L (26.7)	P (2.2)	FL (6.7)	5.61	16.2	47.2	81.5	56.9
Ribonucleases	21	67	57	0	S (23.4)	W (3.3)	TS (4.1)	15.9	38	56.4	58.2	81.8
Somatotropin	25	76	44	13	S (20.0)	W (0.1)	ED (5.0)	21.6	22.9	55.2	62.8	80.4
Kappa-casein	33	92	32	22	V(26.4)	C (0.6)	VA(9.6)	12.7	12.9	48.7	80.6	48.8

*percent/100 MYr; Divergence rates (second column of the table) for protein BLOCKs (first column) are taken from (Marks, 1988; Dayhoff and Schwartz, 1978). LDH: Lactate dehydrogenase; G3PDH: Glyceraldehyde 3-phosphate dehydrogenase; €Dominant pair indicates the hetero-pair type whose observed frequency is maximum for Block.												

**Table 2:** Positional analysis of BLOCKs for invariant line (only one type of residue), conserved position (Shannon entropy ≤1.0) and type of amino acid classes (such as HB, Ac, Bs, PC, ST, HB+HL and PU+PC). Normalized values (in %) are presented for comparison among BLOCKs

Blocks	Dv Rate	INV	CONV	HB	Ac	Bs	PC	ST	HB+HL	PU+PC
Ubiquitin	0.1	7	43.7	11.3	-	2.8	-	-	62	16.9
G3PDH	2.2	15.2	39	19.5	0.6	0.3	2.8	1.5	56	4
LDH	3.4	3.6	21.5	8.8	0.4	-	0.4	0.4	84.3	2.2
Acid-protease	9	1.9	24.8	5.7	-	-	1	-	88.6	2.9
Hemoglobin	12	-	29	6.5	-	-	-	-	93.5	-
Ribonuclease	21	-	10.5	5.3	-	-	2.6	-	89.5	2.6
Somatotropin	25	14.4	39.4	7.5	2.5	3.1	0.6	0.6	60	11.3
Kappa-casein	33	1	64.6	15.2	2	-	-	-	76.8	5.1

INV Invariant position; CONV Conserve position; HB position contains only hydrophobic amino acids; Similarly Ac acidic, Bs basic, PC Polar charge, ST serine plus threonine; HB+HL position contains hydrophobic and hydrophilic amino acids; similarly PU+PC polar uncharged and polar charged; - absent.										

## References

[1] Betts MJ, Russell RB. (2007). Bioinformatics for Genet.

[2] Iengar P. (2012). Nucleic Acids Res.

[3] Ng PC, Henikoff S. (2006). Annu Rev Genomics Hum Genet.

[4] Henikoff S, Henikoff JG. (1992). Proc Natl Acad Sci.

[5] Hendry AP, Kinnison MT. (2001). Genetica.

[6] Marks J. (1988). Columbia University Press New York.

[7] Dayhoff MO, Schwartz RM. (1978). In Atlas of protein sequence and structure.

[8] Dickerson RE. (1971). J Mol Evol.

[9] dos Reis M (2016). Nat Rev Genet.

[10] Tourasse NJ, Li WH. (2000). Mol Biol Evol.

[11] Marini NJ (2010). PLoS Genet.

[12] Baxevanis AD, Ouellette BF. (2004). John Wiley & Sons, New Jersey.

[13] Larkin MA (2007). Bioinformatics.

[14] Altschul SF (1990). J Mol Biol.

[15] Gabaldón T, Koonin EV. (2013). Nat Rev Genet.

[16] Ajawatanawong P, Baldauf SL. (2013). BMC Evol Biol.

[17] Chun S, Fay JC. (2009). Genome Res.

[18] UniProt Consortium.  (2008). Nucleic Acids Res.

[19] Banerjee S (2015). Bioinformation.

[20] Shannon CE. (2001). ACM SIGMOBILE Comput Com Rev.

